# ICTV Virus Taxonomy Profile: *Discoviridae* 2023

**DOI:** 10.1099/jgv.0.001926

**Published:** 2023-12-07

**Authors:** Jens H. Kuhn, Scott Adkins, Katherine Brown, Juan Carlos de la Torre, Michele Digiaro, Holly R. Hughes, Sandra Junglen, Amy J. Lambert, Piet Maes, Marco Marklewitz, Gustavo Palacios, Takahide Sasaya, Yong-Zhen Zhang, Massimo Turina

**Affiliations:** ^1^​ Integrated Research Facility at Fort Detrick, National Institute of Allergy and Infectious Diseases, National Institutes of Health, Fort Detrick, Frederick, MD 21702, USA; ^2^​ United States Department of Agriculture, Agricultural Research Service, US Horticultural Research Laboratory, Fort Pierce, FL 34945, USA; ^3^​ Division of Virology, Department of Pathology, Addenbrookes Hospital University of Cambridge, Cambridge CB2 0QN, UK; ^4^​ Department of Immunology and Microbiology IMM-6, The Scripps Research Institute, La Jolla, CA 92037, USA; ^5^​ CIHEAM, Istituto Agronomico Mediterraneo di Bari, 70010 Valenzano, Italy; ^6^​ Centers for Disease Control and Prevention, Fort Collins, CO 80521, USA; ^7^​ Institute of Virology, Charité-Universitätsmedizin Berlin, Corporate Member of Freie Universität Berlin, Humboldt-Universität zu Berlin, and Berlin Institute of Health, Berlin 10117, Germany; ^8^​ KU Leuven, Rega Institute, Zoonotic Infectious Diseases Unit, 3000 Leuven, Belgium; ^9^​ FIND, 1202 Geneva, Switzerland; ^10^​ Department of Microbiology, Icahn School of Medicine at Mount Sinai, New York, NY 10029, USA; ^11^​ Institute for Plant Protection, National Agriculture and Food Research Organization, Tsukuba, Ibaraki 305-8517, Japan; ^12^​ School of Life Sciences and Human Phenome Institute, Fudan University, Shanghai 201052, PR China; ^13^​ Institute for Sustainable Plant Protection, National Research Council of Italy (IPSP-CNR), 10135 Torino, Italy

**Keywords:** *Discoviridae*, ICTV Report, orthodiscovirus, taxonomy

## Abstract

*Discoviridae* is a family of negative-sense RNA viruses with genomes of 6.2–9.7 kb that have been associated with fungi and stramenopiles. The discovirid genome consists of three monocistronic RNA segments with open reading frames (ORFs) that encode a nucleoprotein (NP), a nonstructural protein (Ns), and a large (L) protein containing an RNA-directed RNA polymerase (RdRP) domain. This is a summary of the International Committee on Taxonomy of Viruses (ICTV) Report on the family *Discoviridae*, which is available at ictv.global/report/discoviridae.

## Virion

Unknown.

## Genome

**Fig. 1.**
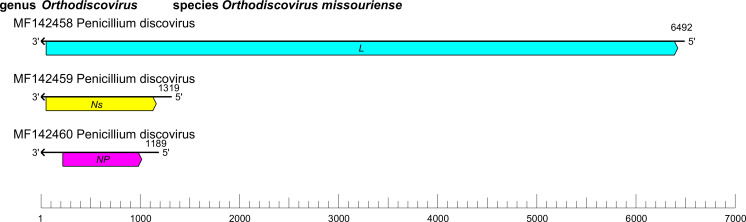
Genome organisation of Penicillium discovirus. ORFs are indicated as boxes, coloured according to the predicted protein function (*L*, large protein gene; *NP*, nucleoprotein gene; *Ns*, nonstructural protein gene).

The genome of discovirids comprises three RNA segments (small [S], medium [M], and large [L]) of linear negative-sense RNA with a total length of 6.2–9.7 kb (S segment: about 1.1–1.2 kb; M segment: about 1.7–1.9 kb; and L segment: about 3.4–6.5 kb) (Table 1). Each segment contains at least one ORF that encodes either an NP, an Ns or an L protein containing an RNA-directed RNA polymerase (RdRP) domain [1–3] (Fig. 1).

**Table 1.** Characteristics of members of the family *Discoviridae*

Example	Penicillium discovirus (S: MF142460; M: MF142459; L: MF142458), species *Orthodiscovirus missouriense*, genus *Orthodiscovirus*
Virion	Unknown
Genome	6.2–9.7 kb of trisegmented negative-sense RNA
Replication	Unknown
Translation	Unknown
Host range	Peronosporaceaen stramenopiles, eurotiomycete and sordariomycete fungi
Taxonomy	Realm *Riboviria*, kingdom *Orthornavirae*, phylum *Negarnaviricota*, class *Ellioviricetes*, order *Bunyavirales*; the family includes the genus *Orthodiscovirus* and several species

## Replication

Unknown.

## Taxonomy

**Fig. 2.**
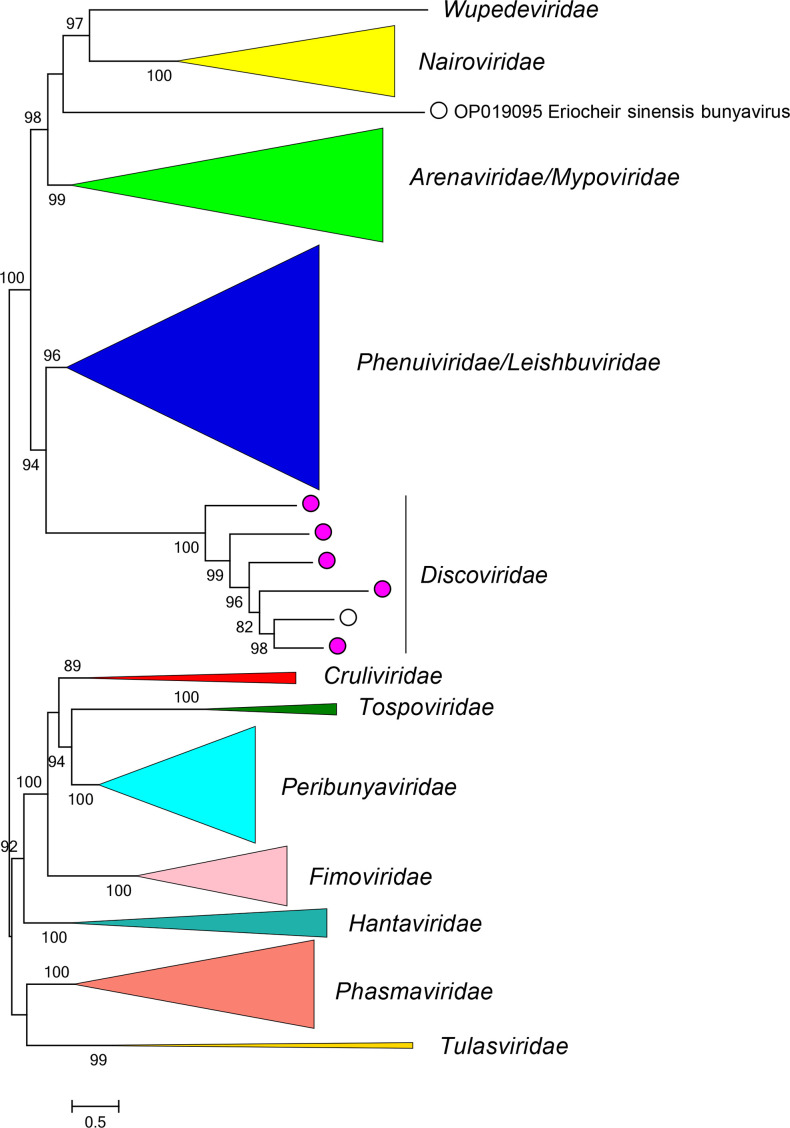
Phylogenetic relationships of viruses in the family *Discoviridae*. Branches for other families are collapsed. Numbers at nodes indicate bootstrap support >70 %. Full details of the virus sequences and methods used are available in the full ICTV Report on the family *Discoviridae.*

Current taxonomy: ictv.global/taxonomy. Discovirids are most closely related to arenavirids, leishbuvirids, mypovirids, nairovirids, phenuivirids, and wupedevirids [4] (Fig. 2). The family includes the genus *Orthodiscovirus* and >4 species; numerous discovirus-like sequences have also been found in sequence-read archives [5]. Like most other bunyavirals, Discovirids (i) have multisegmented, negative-sense single-stranded RNA genomes; (ii) encode proteins with high sequence identity to proteins of other bunyavirals, (iii) and have five conserved motifs (A–E) in their RdRP domain.

## Resources

Full ICTV Report on the family *Discoviridae*: ictv.global/report/discoviridae.
